# Different prognostic value of circulating and disseminated tumor cells in primary breast cancer: Influence of bisphosphonate intake?

**DOI:** 10.1038/srep26355

**Published:** 2016-05-23

**Authors:** Sabine Kasimir-Bauer, Katharina Reiter, Bahriye Aktas, Ann-Kathrin Bittner, Stephan Weber, Thomas Keller, Rainer Kimmig, Oliver Hoffmann

**Affiliations:** 1 Department of Gynecology and Obstetrics, University Hospital Essen, University of Duisburg-Essen, Hufelandstrasse 55, D-45122 Essen, Germany; 2 ACOMED Statistik, Fockestr. 57, D-04275 Leipzig, Germany

## Abstract

Disseminated tumor cells (DTCs) in the bone marrow (BM) and circulating tumor cells (CTCs) in blood of breast cancer patients (pts) are known to correlate with worse outcome. Here we demonstrate a different prognostic value of DTCs and CTCs and explain these findings by early clodronate intake. CTCs (n = 376 pts) were determined using the *AdnaTest BreastCancer* (Qiagen Hannover GmbH, Germany) and DTCs (n = 525 pts) were analyzed by immunocytochemistry using the pan-cytokeratin antibody A45-B/B3. Clodronate intake was recommended in case of DTC-positivity. CTCs were detected in 22% and DTCs in 40% of the pts, respectively. DTCs were significantly associated with nodal status (p = 0.03), grading (p = 0.01), lymphangiosis (p = 0.03), PR status (p = 0.02) and clodronate intake (p < 0.0001), no significant associations were demonstrated for CTCs. CTCs significantly correlated with reduced PFS (p = 0.0227) and negative prognostic relevance was predominantly related to G2 tumors (p = 0.044), the lobular (p = 0.024) and the triple-negative subtype (p = 0.005), HR-negative pts (p = 0.001), postmenopausal women (p = 0.013) and patients who had received radiation therapy (p = 0.018). No prognostic significance was found for DTCs. Therefore early clodronate intake can improve prognosis of breast cancer patients and CTCs might be a high risk indicator for the onset of metastasis not limited to bone metastasis.

Despite major improvements in diagnosis and treatment, about 30% of primary breast cancer patients show a relapse of the disease years after first diagnosis which is explained by micrometastatic spread to the bone marrow (BM) by disseminated tumor cells (DTCs) being present in up to 40% of the patients^1^. After the publication of three large cohorts of primary breast cancer patients, the presence and persistence of DTCs during recurrence–free follow-up has been widely accepted as an independent prognostic marker with regard to increased risk for progression free survival (PFS) and overall survival (OS)^2^,^3^,^4^. However, although DTCs have even been used as a monitoring tool for treatment in primary breast cancer BM aspiration is very invasive and less accepted by patients due to pain and discomfort^5^. In this regard, increasing evidence suggested that circulating tumor cells (CTCs) in blood could be a useful biomarker to estimate risk for recurrence in breast cancer. The prognostic significance of these cells has already been demonstrated by different groups^6^,^7^,^8^ and very recently, the independent prognostic relevance of CTCs, both before and after adjuvant chemotherapy, has been demonstrated in an impressive cohort of 2026 primary breast cancer patients participating in a large prospective clinical trial^9^.

Phenotyping of both cell types in primary breast cancer has demonstrated that a proportion of DTCs and CTCs are present in a non-proliferative state and have stem-cell like characteristics, which may explain resistance to conventional chemotherapeutic drugs^10^,^11^,^12^,^13^. Furthermore, a discordant receptor status between the primary tumor and these cells with regard to the human epidermal growth factor receptor 2 (HER2) and the estrogen receptor (ER) has been demonstrated which might lead to uneffective trastuzumab and/or antihormonal treatment^14^,^15^,^16^. Thus, alternative, chemotherapy-as well as receptor independent therapies are urgently needed to eradicate minimal residual disease.

One approach targeting these cells might be the use of bisphosphonates (BPs). In this regard, the intake of clodronate has been shown to increase OS, reduce the frequency of skeletal complications as well as the incidence and number of new bony and visceral metastases in women with breast cancer who were at high risk for distant metastases^17^,^18^. Applying zoledronic acid (ZOL) in patients undergoing adjuvant hormonal therapy (ABCSG-12, ZO-FAST) resulted in a prolonged PFS^19^,^20^,^21^. In contrast, the AZURE trial (ZOL added to adjuvant therapy), only showed an improved OS for patients who were postmenopausal for at least 5 years^22^. However, analysing the data of 18206 women with BP intake over a period of two to five years, a highly significant reduction in local and distant recurrence, bone recurrence and breast cancer mortality was confirmed for postmenopausal women, whereas no effect could be shown for premenopausal women^23^.

Up to now, four small pilot studies have demonstrated that ZOL as well as clodronate contributed to eradicate DTCs, even after years of first diagnosis^24^,^25^,^26^,^27^. In a cohort of 394 patients, after a seven year follow-up, we recently demonstrated that DTCs were not associated with worse prognosis and speculated that this result was associated with the recommendation of early clodronate intake in case of DTC-positivity^28^. However, clodronate intake could only be confirmed in about 46% of DTC-positive patients whereas in the other patients, clodronate intake was unknown.

In the current study, we evaluated DTCs and CTCs in a cohort of 525 primary breast cancer patients diagnosed between 2004 and 2009 with a median follow up of five years and interviewed all DTC-positive patients for clodronate intake. Here we demonstrate a different prognostic value of DTCs and CTCs with regard to PFS which probably can be explained by early intake of clodronate according to DTC status at primary diagnosis.

## Materials

### Patient population and patient characteristics

The study was conducted at the Department of Gynecology and Obstetrics in Essen. In total, 525 primary, non-metastatic breast cancer patients with first diagnosis between Feb 2004 and Dec 2009, in an adjuvant setting, have been evaluated. Patients’ characteristics at the time of diagnosis are shown in Table 1.

### Study design

We conducted a prospective study, which we analyzed retrospectively at a single institution to determine the prognostic value of DTCs in the BM and CTCs in blood of patients with primary, non-metastatic breast cancer in an adjuvant setting. The median follow-up time was 58.6 months (range: 0 to 117.8 months).

Therapeutic strategy for BP intake: Since 1997, all DTC-positive patients, presenting with primary diagnosis of breast cancer in our clinic, were recommended an additional therapy with oral clodronate (2 × 520 mg per day for at least two years), based on the publication of Diel *et al.*^17^, in the New Engl J Med^17^. In a first analysis of 400 patients, diagnosed between 1998 and 2003, no prognostic impact of DTCs could be documented^28^ and a small pilot study demonstrated a positive effect of ibandronate treatment (50 mg per day) on the eradication of DTCs, still present 2–10 years after primary diagnosis^25^. Since the intake of clodronate was only known for a subgroup of patients and, furthermore, depended on the doctor in charge outside our clinic, we here enquired whether our recommendation had been followed interviewing patients (doctors) to evaluate the impact of clodronate on the eradication of DTCs. 15 DTC negative patients were treated with ZOL according to ABCSG-12 data and ZO-FAST study^19^,^21^.

### Eligibility criteria

The eligibility criteria were: histologically proven breast cancer, BM aspiration at time of primary diagnosis, no severe uncontrolled co-morbidities or medical conditions, no further malignancies at present or in history, completion of adjuvant treatment according to guidelines^29^ including adjuvant chemotherapy (anthracyclines, 5-fluorouracil, taxanes, cyclophosphamide), anti-hormonal therapy in case of hormone responsive tumors (tamoxifen or an aromatase inhibitor), trastuzumab in case of HER2-positivity (after FDA approval in November 2006) and radiotherapy. Patients treated with neoadjuvant chemotherapy were excluded.

### Selection and detection of DTCs

Between 10 and 20 ml BM were aspirated from the anterior iliac crests of all primary breast cancer patients at the beginning of surgery of the primary tumor, before start of any therapy and processed within 24 hours. BM tumor cell isolation and detection have been described elsewhere^15^. Briefly, BM cells were isolated from heparinized BM (5000 U/ml BM) by Ficoll-Hypaque density gradient centrifugation (density 1.077 g/mol; Pharmacia, Freiburg, Germany) at 400x g for 30 min. Slides were analyzed for DTCs by immunocytochemistry using the pan-cytokeratin antibody A45-B/B3. Microscopic evaluation of the slides was carried out using the ARIOL system (Applied Imaging).

### Selection, detection and evaluation of CTCs

Two × 5 ml EDTA blood were collected from 376 patients for the isolation of CTCs before the application of therapeutic substances and before surgery with an S-Monovette^®^ (Sarstedt AG & Co.) and stored at 4 °C until further examination. The samples were processed immediately or latest 4 hours after blood withdrawal. CTCs were analyzed with the *AdnaTest BreastCancer* (AdnaGen AG, Langenhagen, Germany). Establishment and validation of this assay has been described in detail elsewhere^12^,^15^. Briefly, all samples underwent immunomagnetic enrichment using the AdnaTest *BreastCancerSelect* followed by RNA isolation and subsequent gene expression analysis [EpCAM (GA733-2), MUC-1, HER2] by reverse transcription and Multiplex-PCR in separated tumor cells using the AdnaTest *BreastCancerDetect*. The *AdnaTest BreastCancer* for the evaluation of CTCs is considered positive if a PCR fragment of at least one tumor associated transcript is clearly detected. Visualization of the PCR fragments was carried out with a 2100 Bioanalyzer using the DNA 1000 LabChips (Agilent Technologies) and the Expert Software Package (version B.02.03.SI307) both Böblingen, Germany. Peaks with a concentration of >0.15 ng/μl are positive for the transcripts GA733-2, MUC1 and HER2. Peaks that are not detected at the above setting are negative (concentration of <0.15 ng/μl). The primers generate fragments of the following sizes: GA 733-2: 395 base pairs (bp), MUC1: 293 bp, HER2: 270 bp and actin: 114 bp.

### Immunohistochemical analysis of the primary tumor

For each of the 525 patients, the tumor type, TNM-staging and grading were assessed in the Departments of Pathology of our University Hospital.

### Statistical analysis

Relationship of DTC/CTC positivity with presented parameters has been assessed by logistic regression modelling. Time related event data (PFS/OS) has been evaluated by applying uni- and bivariable Cox-Regression models. Univariable models have been applied to all presented parameters, providing hazard ratio estimates and their 95% confidence intervals [CIs]. Bivariable models were presented including DTCs/CTCs and one additional parameter as well as their interaction term. Besides assessment of statistical significance of bivariable model related parameters’ effects, pairwise subgroups’ specific hazard ratio estimates have been presented directly allowing for quantification of related to parameter constellation in-/decrease in hazard of event.

### Study approval/Informed consent/Accordance

All specimens were obtained after written informed consent from all subjects prior to inclusion in the study and collected using protocols approved by the clinical Ethic committee of the University Hospital Essen (05/2856). All methods were carried out *in accordance with* the approved guidelines.

## Results

### Patients’ characteristics

Clinical data are shown in detail in Table 1. A total of 525 patients were included into the study. The median age of the patients was 60 years, range 27 to 86 years. 329/525 (63%) patients had T1 tumors, 349/525 (66%) were node-negative, most of the patients had a ductal carcinoma and a predominantly poor or moderately differentiated tumor. 64% of the patients were lymph node negative, ER and PR positivity was observed in 82% (430/525) and 74% (388/525) of the tumors, respectively. In 16% (84/525) of the cases, HER2 was overexpressed. Classifying tumors in subtypes based on their ER, PR and HER2 expression, 72% (377/525) of the tumors were ER and/or PR positive and HER2-negative, 12% (61/525) were triple-negative (ER−/PR−/ HER2−) and 5% (24/525) of the tumors only expressed HER2 (ER−/PR−/HER2+).

After a median follow-up time of 58.6 months (range: 0 to 117.8 months), the OS rate was 92% (483/525 patients) and relapses occurred in 12% (65/525 patients) of cases.

### Correlation of DTCs and CTCs with established prognostic markers and outcome

As apparent from Table 1, the detection rate for CTCs was 22% (84/376 patients) and DTCs were detected in 211/525 patients (40%). Whereas no significant associations with clinical parameters were found for CTCs, DTCs were significantly associated with nodal status (p = 0.03), grading (p = 0.01), lymphangiosis (p = 0.03), PR status (p = 0.02) but not with CTC-status (p = 0.25).

**Figure 1:**
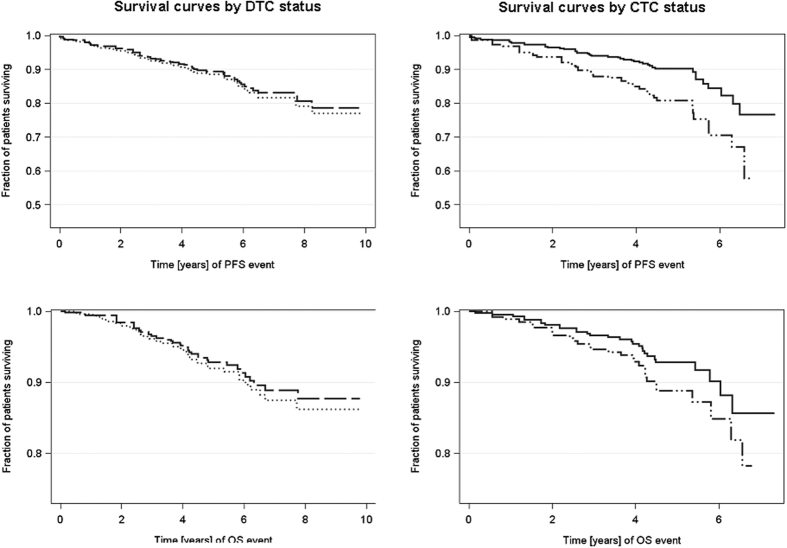
Prognostic significance of CTCs and DTCs with regard to PFS and OS. Kaplan-Meier curves were drawn in order to compare PFS and OS with regard to CTCs in blood and DTCs in the BM. Whereas no significant associations could be shown for DTCs with regard to PFS [P = 0.7215; HR 0.916 (0.564–1.487)] and OS [P = 0.6953; HR 0.885 (0.481–1.630)], CTCs significantly correlated with PFS [P = 0.0227; HR 2.058 (1.106–3.828)] but not with OS [P = 0.267; HR1.588 (0.702–3.595)]. Univariable Cox Regression model-estimated survival curves. Dotted line = DTC negative patients [n = 314], longdashed line = DTC positive patients [n = 211], solid line = CTC negative patients [n = 292], mediumdashdotdot’ed line = CTC positive patients [n = 84].

CTCs significantly correlated with reduced PFS (p = 0.023; Fig. 1) and its negative prognostic relevance was predominantly related to the lobular subtype (p = 0.024), G2 tumors (p = 0.044), the triple-negative subtype (p = 0.005), postmenopausal women (p = 0.013) and patients who had received radiation therapy (p = 0.018) (Table 2). Although no significant correlations with regard to OS could be demonstrated for CTCs alone (p = 0.267; Fig. 1), within triple-negative patients as well as within HR-negative patients, CTC-positive patients had a significantly shorter OS compared to CTC-negative subjects (p = 0.024) (Table 2).

In contrast to CTCs, no prognostic significance could be shown for DTCs with regard to PFS (p = 0.722) and OS (0.695) (Fig. 1). Although no significant correlations with regard to outcome could be demonstrated for DTCs alone, within the subgroup of HER2-positive patients, DTC-negative patients had a significantly shorter PFS (p = 0.003) and OS (p = 0.008) compared to DTC-positive patients.

### Effect of bisphosphonate intake on outcome

**Figure 2:**
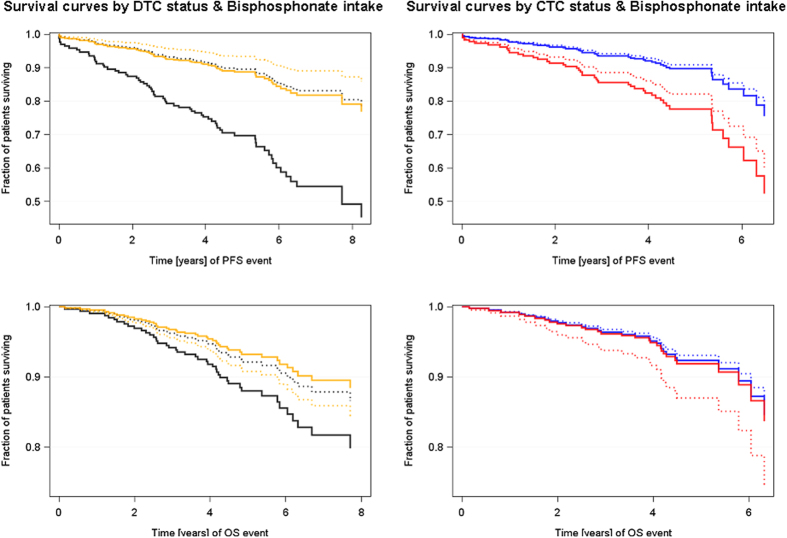
Influence of Bisphosphonates intake on PFS and OS. Kaplan-Meier curves were drawn in order to compare PFS and OS with regard to BP intake in four study groups. DTC-negative patients who had received ZOL seemed to have a worse PFS when compared to DTC-negative patients taking no BPs [p = 0.014; HR 3.268 (1.277–8.364)] as well as to DTC-positive patients who took clodronate [p = 0.025; HR 0.332 (0.126–0.873)]. No significant differences with regard to OS were found between the four groups analyzed. CTC-positive patients who had received clodronate, had a significantly shorter PFS than CTC-negative patients receiving no BPs [p = 0.040; HR 2.629 (1.049–6.591]. No differences in all four groups were shown for OS. Bivariable Cox Regression model-estimated survival curves. Black dotted line = DTC negative patients without BPs [n = 299], black solid line = DTC negative patients with BPs (zoledronic acid) [n = 15], orange dotted line = DTC positive patients without BPs [n = 43], orange solid line = DTC positive patients with BPs [n = 164], blue dotted line = CTC negative patients without BPs [n = 196], blue solid line = CTC negative patients with BPs [n = 94], red dotted line = CTC positive patients without BPs [n = 58], red solid line = CTC positive patients with BPs [n = 25].

Table 1 illustrates BP intake of the patients. Among the 211 DTC-positive patients, 207 could be interviewed for BP intake. The group of 179 patients who had received BP therapy consisted of 164 DTC-positive patients who took clodronate (2 × 520 mg per day) for the duration of two years and 15 postmenopausal DTC-negative patients who had received ZOL (4 mg, twice a year) for the duration of three years. Among the 342 patients who had not taken BPs, 43 were DTC-positive. When the effect of BP intake was correlated with outcome (Fig. 2), patients were stratified into four groups: DTC-positive/BP yes (164 patients), DTC-positive/BP no (43 patients), DTC-negative/BP no (314 patients) and DTC-negative/BP (ZOL) yes (n = 15). As shown in Fig. 2, no significant differences with regard to PFS were found between the first three groups whereas DTC-negative patients who had received ZOL seemed to have a worse PFS when compared to DTC-negative patients taking no BPs (p = 0.014) as well as to DTC-positive patients who took BPs (p = 0.025). No significant differences with regard to OS were found between the four groups analyzed. When PFS and OS was evaluated in pre- and postmenopausal patients comparing those exposed to BPs to those not exposed to BPs, no differential effect of BPs according to menopausal status could be obtained (Supplementary Table 1a–d).

When this patient stratification was applied for CTCs with regard to PFS, CTC-positive patients who had received BPs, had a significantly shorter PFS than CTC-negative patients receiving no BPs (p = 0.040). Notably, only 34% of the CTC-positive patients were also DTC-positive. No differences in all four groups were shown for OS.

## Discussion

The presence of DTCs in the BM and CTCs in blood of breast cancer patients were shown to provide independent prognostic information in a variety of studies and can be regarded as an early indicator of therapy failure^2^,^3^,^4^,^9^. In our current study, contradictory to previous findings of other groups, we demonstrated no prognostic value of DTCs with regard to PFS and OS and explain these findings by early clodronate intake, according to the presence of DTCs at primary diagnosis. In contrast, CTCs were significantly associated with reduced PFS. Thus, we assume that CTCs might be a high risk indicator for the onset of metastasis not limited to bone metastasis.

The limitation of this study is that we did not perform a randomized study. However, one has to consider that the evaluation of DTCs is a special offer, not belonging to daily clinical routine. Thus, offering this method and leaving DTC-positive patients untreated would have been rejected by our local ethic committee and furthermore, would have resulted in no commitment to this study by the patients.

We detected DTCs in 211 of 525 (40%) of our patients which was significantly associated with positive nodal status, higher grading, lymphangiosis and positive PR status but could not show any prognostic significance with regard to PFS. Although no significant correlations with regard to OS could be demonstrated for DTCs alone, in the subgroup of HER2-positive and DTC-negative patients had a significantly shorter PFS (p = 0.003) and OS (p = 0.008) compared to DTC-positive patients which might be explained by the fact that trastuzumab has not been available for most of our study patients during that time. However, in contrast to DTC negative patients the DTC positives received BPs which might explain their better outcome. The positivity rate of DTCs in our patients is in concordance with what previous authors have already reported, with a diagnostic rate of 20% to 40% regardless of nodal status^1^,^30^. The association between DTC status and standard prognostic markers has been discussed controversially. Although most research groups reported a positive association between DTCs and pathological stage and tumor grade, others have failed to document any correlation of DTCs with established clinical prognostic markers^15^,^31^,^32^,^33^. The fact that we could not demonstrate any prognostic significance between DTCs and PFS as well as OS is in contrary to a variety of published studies. While Braun *et al.*, confirmed in his large pooled analysis that DTC detection at the time of primary diagnosis independently predicted an unfavorable outcome with level 1 evidence, others have confirmed that the persistence of DTCs in the BM after adjuvant treatment was an independent marker for reduced PFS and OS^2^,^3^,^16^.

The detection rate for CTCs was 22% (84/376 patients) and was not associated with any other clinicopathological factor. In contrast to DTCs, CTCs significantly correlated with reduced PFS and negative prognostic relevance was predominantly related to the lobular subtype, G2 tumors, the triple-negative subtype, postmenopausal women, HR-negative patients and patients who had received radiation therapy. As already documented for DTCs, the detection rate for CTCs is in accordance with a variety of other published studies using molecular and immunocytological methods^34^,^35^. Furthermore, the prognostic significance of these cells in primary breast cancer before and after adjuvant treatment has already been demonstrated^6^,^7^,^8^,^9^.

Only a few research groups compared the relationship between the presence of DTCs in the BM and CTCs in blood, generally showing a weak correlation. Whereas one study documented a significant congruence between DTCs and CTCs^36^, interestingly, most other groups propose that the prognosis of women with primary breast cancer depends on DTCs rather than CTCs and that CTCs seem to be less prognostic^37^,^38^,^39^. In our study, no significant correlation between DTCs and CTCs was found but, in contrast to other studies, CTCs rather than DTCs were significantly prognostic for early relapse. These findings underline our assumption that CTCs, probably circulating from reservoirs in lung or liver, might be a high risk indicator for already ongoing metastasis not limited to bone metastasis. In this regard, Deng *et al.* analyzed PI3K mutations in DTCs, CTCs and metastases from breast cancer patients and found that single cell analysis of CTCs can reveal genotypic heterogeneity that may change over time, and can show mutational discordance with DTCs and distant metastases^13^. Furthermore, using comparative genomic hybridization and next generation sequencing for the comparison of primary tumors, metastases and CTCs, Heitzer *et al.*, demonstrated that most mutations initially found only in CTCs were also present at subclonal level in the primary tumors and metastases from the same patient^40^.

However, although tumor cell dissemination to the bone occurs in about 30% of the patients, only a minority of DTC-positive patients will develop distant metastasis in the course of the disease. Unfortunately, it is currently not predictable which of these cells will evolve into metastasis. It has been shown that DTCs show a clinically significant biological heterogeneity^41^. By use of comparative genomic hybridization, Mathiesen *et al.*, demonstrated that the frequency of copy number changes of the DTCs revealed similarities with primary breast tumors in more than two third of the analyzed DTCs and similar aberration patterns were visible in DTCs collected at diagnosis and at 3 years relapse-free survival^42^. Whole-genome amplification and subsequent next generation sequencing of two primary breast tumors and corresponding DTCs recently showed comparable whole- arm gains or losses but also clear differences between the primary tumor and DTCs indicating that DTCs underwent further evolution^43^.

In addition, we and others recently demonstrated that a subset of DTCs/CTCs show stem cell character or undergoes epithelial mesenchymal transition (EMT) which might explain why several treatment options are not able to eradicate these cells^10^,^11^,^12^. The fact that the negative prognostic impact of CTCs in our study was mostly related to the triple-negative subtype and patients who had received radiation therapy is in good concordance with our previous studies and can be explained by resistance formation due to metabolism changes in EMT or stem cell like CTCs, demonstrating that CTCs show EMT and tumor stem cell characteristics^12^,^15^. Thus, down regulating targets like ER, PR and HER2, once in circulation, probably makes targeted therapy less effective. Preliminary data of a subgroup of our patients strengthens the assumption that stem cell like CTCs are present among CTCs detected in these patients. Whether these cells predict worse outcome has to be further analyzed in a bigger patient cohort.

The presence as well as the persistence of DTCs and CTCs after therapy clearly indicates a rationale for testing of alternative or secondary treatment options. In this regard, some efforts, including additional chemo, -antibody or BP therapy, have already been published.

### Chemotherapy

In a Norwegian study, patients were treated with docetaxel 100 mg/m^2^, 3qw, 6 courses, if DTC were present 6 months after having completed anthracycline-based chemotherapy. As a consequence, most of the patients experienced disappearance of DTCs with a change to DTC-negativity in 79% of the cases^5^. The success of a switch from anthracycline to docetaxel even if not targeted, might be explained by the different intracellular targets. Whereas anthracycline acts as DNA damaging agent and mRNA synthesis blocker, taxols mitostatic effects result from interactions with microtubuli ant prevents cell division. However, since this study was no randomized trial, other treatments like endocrine and or trastuzumab treatment might have eradicated these cells.

### Targeted therapy

A variety of studies including ours have indicated that HER2 expression on both DTCs and CTCs differed from HER2 expression in the primary tumor and that the expression of HER2 on DTCs and CTCs was correlated with poor prognosis^16^,^44^. Due to this disconcordance, a recently published study indicated that only a minority of patients with HER2-positive DTCs were treated with trastuzumab^16^. In this regard, two pilot studies showed that trastuzumab is able to eliminate these cells^45^,^46^. In the present study, especially for the triple negative subtype, we found a negative correlation for the presence of CTCs and PFS. These findings raise the question, whether a further molecular characterization with regards to ER, PR and HER2 expression on CTCs might be able to provide predictive information of a potential phenotype switch compared to the primary tissue, as already shown by our group^15^ that could lead new therapeutic opportunities.

### Bisphosphonates

Since all targeted therapies can only eliminate DTCs/CTCs presenting the target of interest, receptor independent therapies are highly appreciated to eliminate the residual disease. In this regard, the intake of BPs has been discussed to be successful in eradicating DTCs. In a cohort of 394 patients, diagnosed between 1998 and 2003, we recently demonstrated that DTCs were not associated with worse prognosis and speculated that this result was associated with the recommendation of early clodronate intake in case of DTC-positivity at primary diagnosis^28^. In four small pilot studies, ZOL as well as ibandronate were shown to contribute to eradication of DTCs, even after years of first diagnosis. Rack *et al.*, demonstrated that the administration of 4 mg ZOL per month for the duration of 6 months in patients with persisting DTCs resulted in an elimination of DTCs in 87% of the patients^24^ and Solomayer *et al.* showed a positive effect of ZOL concerning the change of DTCs in the BM of patients with primary non-metastatic breast cancer resulting in a higher number of DTC-negative BM cases in the ZOL group as compared to the control group after 12 months^26^. In the study by Banys *et al.*, primary, DTC-positive breast cancer patients were randomized to treatment with ZOL plus adjuvant systemic therapy or adjuvant systemic therapy alone showing that all ZOL treated patients were DTC-negative after 24 months and that patients with persisting DTCs 12 months after first diagnosis had a significantly shorter OS^27^. We demonstrated that DTCs present in the BM 2–10 years after first diagnosis were successfully eliminated by ibandronate treatment (50 mg per day) for 12 months in all patients^25^. In a retrospective single-center analysis with 3141 early stage breast cancer patients, Hartkopf *et al.* confirmed the prognostic impact of DTCs with regard to DFS and OS and further showed that application of BP treatment was significantly associated with increased DFS and OS in DTC-positive patients, whereas no effect was seen in DTC negative patients^4^. However, there were some differences with regard to our study. The decision, to use BPs was either due to enrolment in studies that included BP therapy or based on the decision of the treating physician. Furthermore, they stated that BPs were mostly used in postmenopausal, hormone receptor positive and DTC positive patients. In our study, BPs were offered in case of DTC-positivity, independent of menopausal status. They further showed that cytotoxic treatment before BM sampling was the strongest risk factor for DTC detection whereas the remaining factors had no impact on DTC status in the multivariate analysis. It is very important to know that these results were due to the inclusion of 391 neoadjuvant patients who were excluded in our study and published separately recently^47^.

However, although these studies impressively documented the antineoplastic effect of BPs on DTCs, big clinical BP studies, especially the AZURE trial, showing an improved survival only for patients who were postmenopausal for at least five years, dampened the euphoria that BPs might be the therapy of choice for eliminating minimal residual disease in all breast cancer patients^22^. With regard to clodronate, the NSABP B-34 trial (patients randomized to receive clodronate, 1.600 mg or placebo for three years) and the GAIN trial (patients randomized to receive oral ibandronate, 50 mg daily for two years) have confirmed the restricted benefit of BPs^48^,^49^. Apart from the ABCSG 12 and the ZOFAST trial, the majority of studies, addressing the antineoplastic effect of BPs, included very heterogenous patient populations, different BPs with different durations of treatment which made difficulties in interpreting the results^19^,^20^,^50^,^51^.

In our patient cohort, no significant differences for DTCs in pre-and postmenopausal women were observed with regard to PFs and OS. This might be due to the small number of patients in these subgroups. Interestingly, a negative prognostic impact with regard to PFS could be demonstrated for CTC-positive, postmenopausal women when compared with CTC-negative women.

Nevertheless, the benefit of BPs in the pre- or perimenopausal setting is very questionable. The meta-analysis of the Early Breast Cancer Trialists´ Colloborative Group had influence on clinical practice, especially for postmenopausal women who showed highly significant reductions in recurrence, distant recurrence, bone recurrence and breast cancer mortality resulting in the decision to treat this patient group with BPs^23^. According to the results of the ABCSG-12 trial, only premenopausal women receiving LHRH antagonists or developing complete ovarian suppression following adjuvant chemotherapy had a benefit from BPs^19^. In our study, the number of patients/events per sub group was too small to draw any conclusions on the missing differential effect of BPs according to menopausal status on PFS and OS.

In conclusion, the limitation of this study is that we did not perform a randomized study. However, one has to consider that the evaluation of DTCs is a special offer, not belonging to daily clinical routine. Thus, offering this method and leaving DTC-positive patients untreated would have been rejected by our local ethic committee and furthermore, would have resulted in no commitment to this study by the patients. Nevertheless, although our control group of 43 patients is too small to draw the final conclusion that the lack of prognostic value of DTCs is likely to be secondary to BPs, we here demonstrate that DTC-positive patients, receiving clodronate at primary diagnosis, have the same prognosis as patients with no DTCs, independent of their menopausal status. Further prospective studies with higher patient numbers will have to answer that question. Interestingly, CTCs might be a high risk indicator for the onset of metastasis not limited to bone metastasis.

## Additional Information

**How to cite this article**: Kasimir-Bauer, S. *et al.* Different prognostic value of circulating and disseminated tumor cells in primary breast cancer: Influence of bisphosphonate intake? *Sci. Rep.* **6**, 26355; doi: 10.1038/srep26355 (2016).

## Supplementary Material

Supplementary Information

## Figures and Tables

**Table 1:** Clinical data of patients.

Total	Total	CTC pos(%)	p-value	Total	DTC pos(%)	p-value
	376	84 (22)		525	211 (40)	
Median age	60 years (range 27–86 years)					
**Tumor size**						
pT1	252	51 (20)	0.37	329	127 (39)	0.07
pT2-4	121				83 (43)	
pT5	3				1 (33)	
**Nodal Status**						
Node negative	256	55 (21)	0.53	349	129 (37)	**0.03**
Node positive	119	29 (24)		174	82 (47)	
**Histology**						
Ductal	289	65 (22)	0.06	397	164 (41)	0.54
Lobular	47	15 (32)		70	24 (34)	
Others	40	4 (10)		58	23 (40)	
**Grading**						
I	75	13 (17)	0.45	94	25 (27)	**0.01**
II	205	50 (24)		277	118 (43)	
III	96	21 (22)		153	68 (44)	
**Lymphangiosis**						
Negative	307	69 (22)	0.92	418	159 (38)	**0.03**
Positive	65	15 (23)		103	51 (50)	
**Haemangiosis**						
Negative	371	84 (23)	0.99	510	205 (40)	0.88
Positive	1	0 (0)		8	3 (38)	
**ER Status**						
Negative	60	14 (23)	0.85	94	44(47)	0.14
Positive	315	70 (22)		430	166 (39)	
**PR Status**						
Negative	74	16 (22)	0.86	136	66 (49)	**0.02**
Positive	301	68 (23)		388	144 (37)	
**Her2 Status**						
Negative	312	68 (21)	0.22	438	170 8399	0.19
Positive	52	15 (29)		84	39 (46)	
**Menopausal Status**						
Premenopausal	45	11 (24)	0.75	72	35 (49)	0.30
Perimenopausal	44	8 (18)		67	26 (39)	
Postmenopausal	287	65 (23)		386	150 (39)	
**Immunhistochemical Subtype**						
(ER−, PR−, Her2−)	40	9 (23)	0.47	61	27 (44)	0.22
(ER−, PR−, Her2+)	12	2 (17)		24	14 (58)	
(ER+ and/ or PR+, Her2−)	281	59 (21)		377	143 (38)	
(ER+ and/ or PR+, Her2+)	41	13 (32)		61	25 (41)	
**Bone marrow Status**						
Negative	243	59 (24)	0.25			
Positive	131	25 (19)				
**Bisphosphonate Intake**						
No	254	58 (23)	0.69	342	43 (13)	**<0.0001**
Yes	119	25 (21)		179	164 (92)	

**Table 2:** Univariable Analysis and Bivariable Cox Regressions.

Univariable Analysis and Bivariable Cox Regressions	CTCs		DTCs	
Univariable Analysis	PFS	OS	PFS	OS
**CTCs**	**2.058 [1.106**–**3.828); p = 0.023**	1.588 [0.702–3.595], p = 0.2671	–	–
**DTCs**	–	–	0.916 [0.564–1.487], p = 0.722	0.885 [0.481–1.630], p = 0.695
**Bivariable Analysis**				
**Histology**				
Ductal Subtype	1.728 [0.822–3.634], p = 0.149	1.405 [0.505–3.905], p = 0.515	0.802 [0.459–1.402], p = 0.439	0.688 [0.326–1.452], p = 0.326
Lobular Subtype	**4.621 [1.227–17.405], p = 0.024**	3.540 [0.705–17.787], p = 0.125	2.011 [0.539–7.502], p = 0.298	3.114 [0.570–17.027], p = 0.190
**Grading**				
**G1**	1.281 [0.143–11.477], p = 0.825	2.588 [0.234–28.567], p = 0.438	2.324 [0.469–11.521], p = 0.302	2.426 [0.342–17.235], p = 0.376
**G2**	**2.353 [1.023–5.410], p = 0.044**	1.540 [0.542–4.379], p = 0.418	0.695 [0.347–1.392], p = 0.305	0.490 [0.201–1.193], p = 0.116
**G3**	1.929 [0.679–5.482], p = 0.218	1.329 [0.274–6.435], p = 0.724	0.915 [0.434–1.928], p = 0.815	1.335 [0.496–3.596], p = 0.567
**IHC-Subtype**				
ER+ and or PR+/HER2−	2.096 [0.949–4.628], p = 0.067	1.466 [0.472–4.553]; p = 0.508	1.126 [0.611–2.074], p = 0.704	1.039 [0.454–2.377], p = 0.929
ER+ and or PR+/HER2+	n.a., no events CTC+	n.a., no events CTC+	0.454 [0.088–2.345], p = 0.346	0.752 [0.125–4.510], p = 0.755
HER2+/ER−/PR−	3.671 [0.694–19.420], p = 0.126	1.128 [0.12–10.206], p = 0.915	**0.123 [0.032–0.481], p = 0.003**	**0.056 [0.007–0.465], p = 0.008**
Triple-negative	**8.565 [1.897–38.661], p = 0.005**	**7.924 [1.315–47.769], p = 0.024**	1.660 [0.446–6.185], p = 0.450	2.229 [0.533–9.331], p = 0.272
**Radiotherapy**				
**(yes)**	**2.434 [1.165**–**5.087],p = 0.018**	2.121 [0.860–5.231], p = 0.103	0.937 [0.526–1.671], p = 0.827	1.165 [0.590–2.300], p = 0.660
**(no)**	1.059 [0.205–5.467], p = 0.945	n.a., no events CTC+	2.113 [0.596–7.494], p = 0.247	0.894 [0.149–5.356], p = 0.903
**Hormone Receptor**				
**(pos)**	1.529 [0.707–3.304], p = 0.281	1.066 [0.353–3.217], p = 0.910	0.950 [0.526–1.715], p = 0.865	0.829 [0.375–1.835], p = 0.644
**(neg)**	**6.910 [2.188–21.823], p = 0.001**	**4.310 [1.150–16.123], p = 0.030**	0.708 [0.293–1.709], p = 0.442	0.889 [0.333–2.370], p = 0.814
**Menopausal status**				
**Post**	**2.270 [1.183–4.453], p = 0.014**	1.850 [0.802–4.266], p = 0.149	0.722 [0.410–1.270], p = 0.258	0.850 [0.433–1.667], p = 0.636
**Prä**	1.187 [0.123–11.453], p = 0.882	n.a., no events CTC+	2.271 [0.601–8.576], p = 0.226	1.194 [0.199–7.165], p = 0.847

n.a. not applicable.

Hazard ratio (p-value); presented HR are estimated for DTC+/CTC + cases within respective subgroup [=row] from Cox regression model.
