# Supplementary material for: Different prognostic value of circulating and disseminated tumor cells in primary breast cancer: Influence of bisphosphonate intake?
Source: Sci Rep. 2016 May 23;6:26355. doi: 10.1038/srep26355 (PMC4876469; doi:10.1038/srep26355)
Supplement: Supplementary Information [file srep26355-s1.pdf]

Different prognostic value of circulating and disseminated tumor cells in primary breast cancer. Influence of bisphosphonate intake?

Sabine Kasimir-Bauer[1], Katharina Reiter[1], Bahriye Aktas[1], Ann-Kathrin Bittner[1], Stephan Weber[2], Thomas Keller[2], Rainer Kimmig[1], *Oliver Hoffmann[1].

Running title: Effect of bisphosphonate intake on minimal residual disease

Table 1a Absolute and relative frequencies of PFS event occurrence by DTC status by menopausal status and bisphosphonate intake

Menopausal status	DTC status	Events	Bisphosphonate intake		
			No (%)	Yes (%)	Total (%)
premenopausal	negative	no event	34 (91,9)	0 (0)	34 (91,9)
		event	3 (8,1)	0 (0)	3 (8,1)
	positive	no event	5 (83,3)	22 (75,9)	27 (77,1)
		event	1 (16,7)	7 (24,1)	8 (22,9)
pre-/perimenopausal	negative	no event	70 (93,3)	3 (100)	73 (93,6)
		event	5 (6,7)	0 (0)	5 (6,4)
	positive	no event	12 (92,3)	39 (81,3)	51 (83,6)
		event	1 (7,7)	9 (18,8)	10 (16,4)
postmeopausal	negative	no event	195 (87,1)	7 (58,3)	202 (93,6)
		event	29 (12,9)	5 (41,7)	34 (6,4)
	positive	no event	27 (90,0)	101 (87,1)	128 (83,6)
		event	3 (10,0)	15 (12,9)	18 (16,4)

Table 1b 1a Absolute and relative frequencies of OS event occurrence by DTC status by menopausal status and bisphosphonate intake

Menopausal status	DTC status	Events	Bisphosphonate intake		
			No (%)	Yes (%)	Total (%)
premenopausal	negative	no event	35 (94,6)	0 (0)	35 (94,6)
		event	2 (5,4)	0 (0)	2 (5,4)
	positive	no event	5 (83,3)	27 (93,1)	32 (91,4)
		event	1 (16,7)	2 (6,9)	3 (8,6)
pre-/perimenopausal	negative	no event	72 (96)	3 (100)	75 (96,2)
		event	3 (4)	0 (0)	3 (3,8)
	positive	no event	12 (92,3)	45 (93,8)	57 (93,4)
		event	1 (7,7)	3 (6,3)	4 (6,6)
postmeopausal	negative	no event	204 (91,1)	10 (83,3)	214 (90,7)
		event	29 (8,9)	2 (16,7)	22 (9,3)
	positive	no event	26 (86,7)	106 (91,4)	132 (90,4)
		event	4 (13,3)	10 (8,6)	14 (9,6)

Table 1c Absolute and relative frequencies of PFS event occurrence by menopausal status for DTC+/CTC- and bisphosphonate intake

Menopausal status	Events	Bisphosphonate intake		
		No (%)	Yes (%)	Total (%)
premenopausal	no event	1 (100)	10 (83,3)	11 (84,6)
	event	0 (0)	2 (16,7)	2 (15,4)
pre-/perimenopausal	no event	6 (100)	20 (87,0)	26 (89,7)
	event	0 (0)	3 (13,0)	3 (10,3)

Table 1d Absolute and relative frequencies of OS event occurrence by menopausal status for DTC+/CTC- and bisphosphonate intake

Menopausal status	Events	Bisphosphonate intake		
		No (%)	Yes (%)	Total (%)
premenopausal	no event	1 (100)	11 (91,7)	12 (92,3)
	event	0 (0)	1 (8,3)	2 (7,7)
pre-/perimenopausal	no event	6 (100)	21 (91,3)	26 (93,1)
	event	0 (0)	2 (8,7)	3 (6,9)
